# Attitude towards Antipsychotic Medications in Patients Diagnosed with Schizophrenia: A Cross-Sectional Study at Amanuel Mental Specialized Hospital, Addis Ababa, Ethiopia

**DOI:** 10.1155/2019/5094017

**Published:** 2019-05-22

**Authors:** Tilahun Kassew, Demeke Demilew, Addis Birhanu, Mesele Wonde, Biks Liyew, Shegaye Shumet

**Affiliations:** ^1^Department of Psychiatry, University of Gondar, Gondar, Ethiopia; ^2^Amanuel Mental Specialized Hospital, Addis Ababa, Ethiopia; ^3^Department of Emergency and Critical Care Nursing, University of Gondar, Gondar, Ethiopia

## Abstract

**Background:**

Poor attitude towards antipsychotic drugs is high, and it is a factor for non-adherence to treatment. This increases the risk of relapse, associated healthcare utilization, and costs. This study aimed to assess attitude towards antipsychotic medication among patients with schizophrenia.

**Objectives:**

The aim of this institution based cross-sectional study was to assess attitude towards antipsychotic medications and associated factors among patients with schizophrenia who attend the outpatient clinics at Amanuel Mental Specialized Hospital, 2018.

**Methods:**

In a cross-sectional study, 393 schizophrenic patients from Amanuel Mental Specialized Hospital were recruited by a systematic random sampling technique. Drug Attitude Inventory (DAI-10) was used to assess attitude, experience, and belief about antipsychotics. Glasgow antipsychotic side effect scale modified version, positive and negative syndrome scale, and Birch wood's insight scale for psychosis were the instruments used to assess the associated factors. Simple and multiple linear regression analysis models were fitted, and the adjusted unstandardized beta (*β*) coefficient at 95% confidence interval was used.

**Results:**

The mean score of attitude towards antipsychotic medications was 6.51 with standard deviation (SD) of 2.22. In multiple linear regression, positive symptoms (*β*= -0.07, 95% CI: (-0.09, -0.05)), negative symptoms (*β*= -0.04, 95% CI: (-0.06,-0.02)), shorter (≤5 years) duration of illness (*β*= -0.39, 95% CI: (-0.63, -0.15)), first generation antipsychotics (*β* = -0.35, 95% CI: (-0.55,-0.14)), having sedation (*β*= -0.28, 95% CI: (-0.52, -0.02)), and extra-pyramidal side effects (*β*= -0.34, 95% CI: (-0.59,-0.09)) were factors negatively associated with attitude towards antipsychotic medication treatment. Insight to illness (*β*= 0.24, 95% CI: (0.20, 0.27) was a factor positively associated with attitude towards antipsychotic medications.

**Conclusion:**

The result suggests that the mean score of participants' attitude towards antipsychotic medications was good. Prevention of side effects particularly due to first generation antipsychotics is necessary.

## 1. Introduction

Schizophrenia is a chronic psychotic disorder with impaired thinking, emotions, and behaviors that affect family relationships, social functioning, and employment. The lifetime prevalence of schizophrenia is 1.0% [1]. Antipsychotic medications are the first line choice of treatment options for patients with schizophrenia. These drugs enhance recovery by controlling symptoms, improving quality of life, regaining basic life functioning, and preventing relapse [2, 3].

Attitude towards antipsychotic medication is the subjective feelings, beliefs, experiences, and opinions of patients with schizophrenia towards the prescribed antipsychotic drugs [4, 5].

Drug compliance in patients with schizophrenia is predicted by the patients' attitude towards medications [6, 7]. Negative attitude towards antipsychotic medication is common in clinical practice with the prevalence ranges from 7.5%-46.7% [8–11]. Up to 75% of those with a negative attitude have non-adherence to antipsychotic drugs, which results in a relapse. The prevalence of relapse due to non-adherence varies from 50% to 92% globally [6, 12]. Patients with schizophrenia are most likely to die early from potentially treatable conditions as a result of non-adherence to prescribed medications [13–15]. Most cases of re-hospitalization of patients diagnosed with schizophrenia are due to non-adherence to medication, which results from a negative attitude towards medication [16–18].

There are several moderating factors for poor attitude towards medication. The moderating factors were younger age [5, 8], male in sex [5, 19], being employed [20–22], urban residence, and living alone, which have a negative relationship with attitude towards medication [22]. Poor attitude towards antipsychotic medication was more common among patients with schizophrenia with a shorter duration of illness (less than five years) [8, 21, 23, 24], later age at onset of illness [25], and having frequent psychiatric hospital admissions [26]. The other important clinical moderating factors for attitude towards medication were the levels of positive symptoms, negative symptoms, and general psychopathology [8, 21, 22, 25, 27, 28], which also have a negative relationship with attitude towards medication. Insight of illness has a positive association with attitude towards medication [5, 25, 29].

Patients who are taking first-generation antipsychotics (FGAs) and depot medications [25, 29] had a more negative attitude towards their treatment. The side-effect burden is often viewed as an important reason for a poor attitude towards antipsychotic medication [20, 30, 31]. Antipsychotic-induced side-effects like dyskinesia, parkinsonism, sexual dysfunction, and sedation were the factors associated with a negative attitude towards antipsychotic medications [9, 21, 28, 30–32]. The severity of side effects is another factor, which affects the attitude towards antipsychotic drugs with a negative relationship [5, 9]. Poor attitude towards antipsychotic medication is also affected by illicit drug use as a result of worsening psychotic symptoms, which leads to poor outcome of the illness [33, 34].

In our clinical observations, many schizophrenic patients discontinue their prescribed medications and re-hospitalized due to relapse of the illness, which result in a high cost for a health care system. Different studies [6, 12] have showed that more than half of patients with schizophrenia have non-adhere to antipsychotic drugs due to their negative attitude towards the medication. Even though attitude has an impact on antipsychotic medication compliance, there is no study showing attitude of patients diagnosed with schizophrenia towards antipsychotic medication in Ethiopia. So identifying attitude of patients diagnosed with schizophrenia towards antipsychotic medication is important for controlling psychotic symptoms, decreasing the burden of relapse, and regaining basic life functioning, which all contributes for improving patients' quality of life.

## 2. Methods and Materials

### 2.1. Study Settings and Populations

Institution based cross-sectional study design was conducted among patients diagnosed with schizophrenia who had follow-up at Amanuel Mental Specialized Hospital (AMSH) in Addis Ababa, Ethiopia, between May and June 2018. Systematic random sampling technique was used to recruit 393 study subjects. The sampling fraction (k) was calculated from the average monthly patients with schizophrenia who had visited the hospital divided by sample size, which was 8. The first participant was chosen randomly by lottery method from numbers 1 to 8. Then, every 8th patient was interviewed.

### 2.2. Measurement

The patients' attitude towards antipsychotic medications was assessed with the 10-items Drug Attitude Inventory (DAI-10) 10-Item. The items of the questionnaire are about the benefits and perceived effects of antipsychotic drugs. The patients' response for the questions was true or false and rated as 0 if response for the asked question is incorrect and 1 if response for asked questions is correct. The total score ranges from 0 to 10. Individuals with the total score closer to 0 indicates very poor attitude and those with the total score closer to 10 indicates best possible attitude on DAI-10 [4].

Symptoms of psychosis were measured using Positive and Negative Syndrome Scale (PANSS), which has three subscales: positive symptoms, negative symptoms, and general psychopathology subscales [35]. Insight to illness was measured using Birch wood's Insight Scale for psychosis (BIS). The instrument is used to assess the three areas of insight, insight into the need for treatment, awareness of illness, and the ability to relabeling experiences. BIS is an 8-item self-report scale, which is a 3-point scale with higher scores indicating better insight [36].

The prevalence, type, and severity of antipsychotic side effects were assessed using the 22-item, modified version of Glasgow Antipsychotic Side effect Scale (GASS). Side effects assessed by GASS were sedation/cognition, cardiovascular side effects, extrapyramidal symptoms (EPS), anticholinergic side effects, gastrointestinal, genitourinary side effects, screening of diabetes mellitus, prolactin/endocrine side effects, and weight gain. The extent of side effects is rated from none (0) to everyday (3 points) for questions 1-20 and no (0) and yes (3 points) for questions 21-22. Patients with a total score of 0-12 indicate absent/mild side effects, 13-26 moderate side effects, and over 26 severe side effects [37]. 

Items on socio-demographic factors (age, sex, ethnicity, religion, marital status, educational status, and occupational status) were adopted from different articles.

### 2.3. Data Collection

Structured questionnaires including socio-demographic, illness related, medication related, substance related characteristics, and DAI-10 questionnaires were used to collect the data. Data were collected by four trained data collectors (four mental health professionals) using the Amharic version of the questionnaire for a month. The questionnaire was designed in English and was translated to Amharic, the official language of Ethiopia and back to English, forward and backward translation for its consistency. The training was on introduction to antipsychotic medications, research methods, interviewing skills, sampling and recruitment, and ethical aspects of research.

### 2.4. Data Processing and Analysis

Data were checked for completeness and consistency. A coded variable was entered into EpiData version 3.1, then exported to and analyzed using SPSS version-20. Descriptive statistics (frequency, percent, mean, and standard deviation) were used to summarize the distribution of variables. Assumption test was checked before conducting the regression analysis. Simple linear regression analysis was performed to test an association between attitude towards antipsychotic medication and each independent variable. Variables with p-value ≤0.05 during simple linear regression analysis were selected for further analysis in multiple linear regression analysis and model fitness test (adjusted R^2^) was also checked. Factors associated with the attitude towards antipsychotic medication were expressed as adjusted unstandardized *β* coefficient by employing 95% confidence level. A p-value of < 0.05 was considered as statistically significant.

### 2.5. Ethical Consideration

Ethical approval was obtained from the joint Ethical Review Committee (ERC) of University of Gondar and Amanuel Mental Specialized Hospital. An informed written consent was obtained from the participants. For the individuals younger than 18 years, written informed assent was obtained from their guardians after explaining the purpose of the study. Confidentiality was maintained by omitting their personal identification.

## 3. Results

A total of 393 participants took part in the study, with the response rate of 98.4%. The mean age of the participants was 36.40 (SD, 9.93) ranging from 15 to 65 years. Nearly, half of the study participants, i.e., N=207 (52.7%), were unmarried and 243 (61.80%) were males. Nearly one-third of the study participants, i.e., N=126 (32.1%), attended secondary school education. The majority of participants, N=262 (66.7%), were from the urban area and 285 (72.5%) of the participants were unemployed. Most of the study participants, N=330 (84.7%), were living with their family/supporters and 120 (30.7%) of the participants were currently using substances for non-medical purpose (Table 1).

The mean age of onset for schizophrenia was found to be 26.77 (SD±7.23). In total, 146 (37.15%) participants were included; duration of illness was 6 to 10 years. Regarding their psychiatric hospital admission history, approximately, half of the study participants did not have previous hospital admission. Only 28 (7.1%) patients had co-morbid medical/mental illness including diabetes mellitus, hypertension, HIV/AIDS, depression, and substance use disorder. The mean total score for degree of psychopathology and insight were 73.65 [SD ± 30.48], 8.9 [SD ± 3.8]), respectively (see Table 2).

Regarding the class of antipsychotics, 224 (57%) of the patients were on first generation antipsychotics (FGAs) medications. Over a fifth of the participants were on two or more antipsychotics medications and nearly one-third were on depot medications. The majority of them were on <300 mg CPZeq dose category (see Table 3).

**Figure 1:**
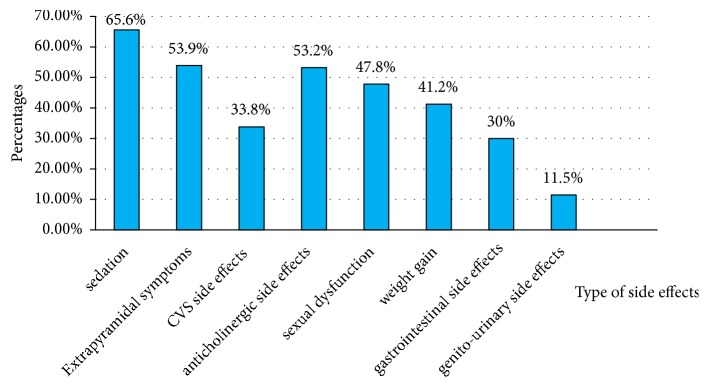
Magnitude of antipsychotics side effects among patients with schizophrenia at AMSH, 2018.

The severity of side effects was rated as mild, moderate, and severe according to Glasgow Antipsychotic Side-effect Scale. About 193 (49.1%) of the participants experienced absent/mild side effects, 123 (31.3%) had moderate side effect, and 77 (19.4%) of them had a severe side effect. The majority of the patients (88.8%) were experiencing at least one side effect due to their medications based on Glasgow antipsychotic side effects rating scale (GASS). Sedation was the most frequent side effect (65.6%) (Figure 1).

### 3.1. Attitude towards Antipsychotic Medications among Patients with Schizophrenia

The mean score of attitude towards antipsychotic medication was 6.51 (95% CI 6.28, 6.74) with the standard deviation of 2.22.

### 3.2. Factors Associated with Attitude towards Antipsychotic Medications

Simple linear regression analysis indicated that residency, duration of illness, onset of age of illness, PANSS positive subscale, PANSS negative subscale, general psychopathology, class of antipsychotic drugs, number of antipsychotic drugs and route, presence of side effect (sedation, extrapyramidal symptoms, sexual dysfunction), and current substance use were factors negatively associated with attitude towards antipsychotic medications with p-value ≤0.05. Insight to illness was a factor positively associated with attitude towards antipsychotic medications. Results of multiple linear regressions showed that higher PANSS positive and negative score, shorter duration (≤5 years) of illness, having poor insight to illness, FGAs, having sedation, and extrapyramidal symptoms were factors associated with negative attitude towards antipsychotic medications.

Attitude towards antipsychotic medications is negatively associated with positive and negative symptoms, shorter duration of the illness, treated with FGAs, presence of sedation, and extrapyramidal symptoms, whereas attitude towards antipsychotic medications is positively associated with insight to illness (Table 4).

## 4. Discussion

Antipsychotic medications are the treatment options for patients diagnosed with schizophrenia. They enhance control for symptoms, prevent relapse, help patients regain basic life functioning, and improve quality of life [2, 3]. For these positive outcomes of antipsychotics, patients' adherence to their medication is necessary [38]. Patients' attitude towards their medication may significantly affect the subjective response to anti psychotics. Studies showed [6, 12] that nearly three-fourth of patients with negative attitude towards their medication have non-adherence to antipsychotic drugs. Our findings indicate that the majority of the respondents had a positive attitude towards antipsychotic medication with mean score of 6.51 (95% CI= 6.27, 6.74). The proportion of those who had a positive attitude towards antipsychotic medication was 51.9%. If the DAI represents an indirect indication for compliance, then the level of non-compliance in our sample was 48.1%. It indicates that nearly half of patients with schizophrenia had non-compliance to their medication. This finding is supported by Nigeria's study with mean score of 6.71 [20]. In the current study, we had a mean higher drug attitude score in patients, as compared to a study conducted in Bulgaria, South Korea, Spain, and USA [24, 27, 39, 40]. The possible reason for the variation might be attributed to the approach to the summation of the items, sample size difference, the study design, socio-cultural differences, and difference between participants. In this study, the approach of scoring of the items was by giving 0 and 1 values, which result in the sum total ranges of 0-10, whereas in Bulgaria, South Korea, and USA -1 and/ 1 values were used for the alternatives and the sum total ranges of -10 to10s. So this finding is supposed to increase the mean score of attitude towards antipsychotic medications. The sample size was higher in this study compared to that of Bulgaria, South Korea, and Spain. The USA study was a large multi-center prospective study whereas in our study, we used a cross-sectional study design, which may have resulted in the mean score difference. In the Spanish study, the participants were hospital discharged patients. But in this study, the participants were patients with schizophrenia on a follow-up, which may have increased the mean score.

Regarding predictor variables, shorter (≤5 years) duration of illness was significantly associated with a negative attitude towards antipsychotic medication. The possible reason might be when duration of illness is short, patients might have poor insight about their illness and in need for treatment, which results in negative attitude towards their medication. This finding was consistent with previous studies [8, 21, 23–25]. The degree of psychopathology was negatively associated with attitude towards antipsychotic medications. Patients with severe positive symptoms and negative symptoms had an association with a poor attitude towards antipsychotic drugs. The possible reason might be due to the patients' delusions, hallucinations, suspiciousness, hostility, and withdrawal behaviors, which may increase the likelihood of a poor attitude toward medication [25]. This result is in agreement with other studies [20, 21, 25, 27].

Insight to the illness had a positive association with the attitude towards antipsychotic medications treatment. If patients believe that they have a mental illness, aware of the benefit of treatment and of the social consequences of the disorder, they would have better subjective feelings and attitude towards the treatment. This finding was consistent with other studies [5, 20, 25, 28, 29, 32], which showed that negative subjective attitude towards drugs was associated with less insight.

In our study, patients treated with FGAs have a lower score of attitude towards antipsychotic medications as compared to patients on SGAs antipsychotic treatment. In fact, this might be due to the perception that these medications are less effective against negative symptoms and more noxious [41], which may result in a poor attitude towards their medications. This finding was in line with other studies [25, 26, 42, 43]. Sedation side effect was significantly associated with poor attitude to antipsychotic medications. Sedation may contribute to irregular drug indigestion, poor control, and increase in psychopathology, which may lead to a decrease in subjective awareness of well-being and patients' subjective need to take medications [44]. This finding was similar to other studies [9, 20, 32, 44, 45]. In addition, this study showed that having EPS symptoms was significantly associated with a poor attitude towards antipsychotic drugs among patients with schizophrenia. The possible reason might be that many patients experience antipsychotic drugs as unpleasant and something bad that they would prefer to avoid it [46]. This finding was supported by other studies [9, 20, 31].

There were several limitations. The participants were stable patients attending the clinic regularly, who were motivated to participate in the study. Patients with a negative attitude to drugs that usually stop attended clinic early might be underrepresented. The other limitation is the cross-sectional nature of the study. Since we were using cross-sectional study design, it is difficult to assess attitude towards medication in the acute phase of the illness and to evaluate if there are changes in attitude towards medication in different phases of illness.

## 5. Conclusion

Patients with schizophrenia have a good mean score of attitude towards antipsychotic medications. The presence of symptoms (positive and negative), having poor insight into the illness, shorter duration of illness, treated with FGAs, and presence of side-effects like sedation and EPS were factors significantly associated with a negative attitude towards antipsychotic medications. The program designers and the clinicians should incorporate these factors to improve the patient's attitude towards medications. Prevention of side effects particularly due to first generation anti psychotics is necessary.

## Figures and Tables

**Table 1:** Socio-demographic and substance related characteristics of patients with schizophrenia at AMSH, 2018 (n=393).

Variables		Frequency	Percent (%)
Sex	Male	243	61.8
	Female	150	38.20

Marital status	Unmarried	207	52.7
	Married	122	31
	Divorced	38	9.7
	Others	26	6.6

Educational status	Unable to read and write	49	12.5
	Primary school	123	31.3
	Secondary school	126	32.1
	Diploma and above	95	24.2

Job status	Unemployed	285	72.5
	Employed	108	27.5

Living arrangement	Living alone	60	15.3
	Living with family/ supporters	330	84.7

Residence	Urban	262	66.7
	Rural	131	33.3

Current substance use	Yes	120	30.5
	No	273	69.5

Type of substances	Khat chewing	87	22.1
	Alcohol use	56	14.2
	Cigarette smoking	76	19.3
	Other substances	10	2.5

Age		Mean= 36.4	SD= 9.93

Others= separated +widowed, other substances= Marijuana, shisha, drugs.

**Table 2:** Clinical characteristics of patients with schizophrenia on follow-up at AMSH, 2018 (n=393).

Variables	Categories	Frequency	Percent (%)
Duration of illness	1-5 years	109	27.7
	6-10 years	146	37.1
	>10 years	138	35.2

Psychiatric hospital admission	No admission	196	49.9
	1-5 times	162	41.2
	≥6 times	35	8.9

Co-morbid chronic medical/ mental disorder	Yes*∗*	28	7.1
	No	365	92.9

	*Ranges*	*Mean *	*SD*

Age of onset of the illness in years	12-52	26.77	7.23

PANSS total score	30-142	73.65	30.48
PANSS positive subscale score	7-44	17.51	9.38
PANSS negative subscale score	7-45	19.23	8.890
PANSS general psychopathology Score	16-87	37.18	15.80

Birchwood's insight scale score	0-16	8.9	3.80
Relabeling experiences	0-4	2.09	1.17
Awareness of illness	0-4	1.74	1.14
Awareness of need of Rx	0-8	5.08	2.16

*∗* Diabetes, hypertension, HIV/ AIDS, depression, substance related disorders; Rx= treatment.

**Table 3:** Drug therapy related characteristics among patients with schizophrenia on follow-up at AMSH, 2018 (n= 393).

Variables	Categories	Frequency	Percent (%)
Class of antipsychotics	FGAs	224	57
	SGAs	123	31.3
	FGAs+ SGAs	46	11.7

No of antipsychotic drug	Single	311	79.1
	Two or more	82	20.9

Route of use	Per mouth	274	69.7
	Parenteral	42	10.68
	Combined route	77	19.59

Frequency	Once	274	69.7
	Twice a day	119	30.3

CPZeq dose	<300mg	232	59.0
	300-600mg	61	15.5
	601-1000mg	45	11.5
	>1000mg	55	14.0

Any adjuvant drug	No	315	80.2
	Yes*∗∗∗*	78	19.8

Note; *∗∗∗* Amitriptyline, Na+ valproate, fluoxetine, bezhexol, benzodiazepines Combined route= per mouth and parenteral, FGAs= First Generation Antipsychotics; SGAs= Second-Generation Antipsychotics.

**Table 4:** Factors associated with attitude towards antipsychotic medications during multiple linear regression analysis (n=393).

Variables	Crude unstandardized *β* coefficient (95% CI)	Adjusted unstandardized *β* coefficient (95% CI)
Residence		
Rural	0	0
Urban	-0.59 (-1.06, -0.12)	-0.10 (-0.29, 0.11)

Age of onset of illness	0.03 (0.002, 0.060)	0.02 (-0.20, 0.24)

duration of illness		
>10 years	0	0
5-10 years	0.36 (-0.10, 0.82)	-0.08 (-0.31, 0.15)
≤5 years	-2.49 (-2.92, -2.05)	-0.39 (-0.63, -0.15)*∗*

PANSS positive score	-0.2 (-0.209, -0.181)	-0.07 (-0.09, -0.05)*∗∗∗*

PANSS negative score	-0.19 (-0.21, -0.17)	-0.04 (-0.06, -.02)*∗∗∗*

PANSS general psychopathology score	-0.09 (-0.10, -0.08)	-0.004 (-0.01, 0.004)

BIS total score	0.49 (0.46, 0.52)	0.24 (0.20, 0 .27 )*∗∗∗*

Class of drugs		
SGAs	0	0
FGAs+ SGAs	-0.58 (-1.19, 0.03)	-0.17 (-0.47, 0.13)
FGAs	-1.89 (-2.30, -1.48)	-0.35 (-0.55, -0.14)*∗∗*

No of Antipsychotics		
Single	0	0
≥2	-1.64 (-2.16, -1.11)	-0.15 (-0.42, 0.12)

Route of use		
Oral	0	0
Parenteral	-1.36 (-1.83, -0.89)	0.02 (-0.21, 0.25)

Sedation side effects		
No	0	0
Yes	-2.59 (-2.99, -2.20)	-0.28 (-0.52,-0.02)*∗*

EPS symptoms		
No	0	0
Yes	-2.59 (-2.96, -2.23)	-0.34 (-0.59, -0.01)*∗∗*

Sexual dysfunction		
No	0	0
Yes	-0.75 (-1.19, -0.31)	0.01 (-0.19, 0.20)

Current substance use		
No	0	0
Yes	-1.54 (-2.00, -1.08)	0.02 (-0.20, 0.24)

Note; *∗* p<0.05, *∗∗* p< 0.01, *∗∗∗* p< 0.001

Adjusted R^2^= 79.9%, F-test p-value<0.001.

## Data Availability

The data used to support the findings of this study are available from the corresponding author upon request.
